# White Matter Microstructure Alterations in Older Adults With Dyslipidemia Associated With Cognitive and Locomotor Dysfunction Evaluated Using Neurite Orientation Dispersion and Density Imaging

**DOI:** 10.1002/brb3.70526

**Published:** 2025-05-28

**Authors:** Zaimire Mahemuti, Christina Andica, Koji Kamagata, Kaito Takabayashi, Wataru Uchida, Sen Guo, Takashi Arai, Hiroki Tabata, Hitoshi Naito, Yoshifumi Tamura, Ryuzo Kawamori, Hirotaka Watada, Shigeki Aoki

**Affiliations:** ^1^ Department of Radiology Juntendo University Graduate School of Medicine Bunkyo‐ku Tokyo Japan; ^2^ Faculty of Health Data Science Juntendo University Urayasu Chiba Japan; ^3^ Sportology Center, Juntendo University Graduate School of Medicine Bunkyo‐ku Tokyo Japan; ^4^ Department of Metabolism & Endocrinology Juntendo University Graduate School of Medicine Bunkyo‐ku Tokyo Japan

**Keywords:** cognitive function, diffusion MRI, diffusion tensor imaging, dyslipidemia, locomotor function, neurite orientation dispersion and density imaging

## Abstract

**Introduction:**

Diffusion tensor imaging (DTI) studies have shown white matter (WM) microstructural alterations in individuals with dyslipidemia; however, DTI indices are not specific to WM pathology. However, neurite orientation dispersion and density imaging (NODDI) provides more specific measurements of WM microstructure. This study aimed to evaluate dyslipidemia‐related WM microstructure alterations and their association with cognitive and motor functions using NODDI.

**Methods:**

The DTI and NODDI metrics were analyzed through tract‐based spatial statistics between 24 older adults with dyslipidemia (low‐density lipoprotein ≥140 mg/dL, high‐density lipoprotein <40 mg/dL, and triglyceride ≥150 mg/dL, or under treatment) and 18 healthy control participants (HCs). Partial correlation tests were performed between diffusion magnetic resonance imaging measures and lipid profiles, cognitive, or locomotor scores in the dyslipidemia and HC groups separately. WM volumetry between HCs and dyslipidemia groups was also assessed. Age, gender, intracranial volume, and years of education were included as covariates in all analyses. A false discovery rate‐corrected *P* value of <0.05 was considered statistically significant.

**Results:**

Individuals with dyslipidemia exhibited a notably reduced neurite density index (NDI) in several WM areas, including the posterior and superior corona radiata, the body, the genu, and the splenium of the corpus callosum, as well as the bilateral anterior and posterior internal capsule, compared with HCs. In the dyslipidemia group, lower NDI was significantly correlated with lower scores on the stand‐up test and the Japanese version of the Montreal Cognitive Assessment. No significant differences were found in DTI metrics or WM volumes between dyslipidemia individuals and HCs.

**Conclusion:**

Our findings suggest that NODDI can serve as a biomarker for assessing WM microstructural alterations in older adults with dyslipidemia. Particularly, NODDI indicates a lower intra‐axonal volume, which may suggest axonal loss associated with dyslipidemia, and correlates with cognitive and locomotor function decline.

## Graphical abstract

This study used NODDI to identify white matter microstructure alterations in older adults with dyslipidemia, revealing significantly reduced neurite density in several brain regions. These reductions were linked to declines in cognitive and motor functions, suggesting that NODDI may serve as a biomarker for dyslipidemia‐related axonal degeneration.

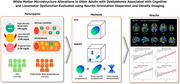

**Abbreviations**
ACR: anterior corona radiata
AD: axial diffusivity
ALIC: anterior limb of internal capsule
AMICO: Accelerated Microstructure Imaging via Convex Optimization
ATR: anterior thalamic radiation
CC: corpus callosum
CCG: cingulum cingulate gyrus
CH: cingulum hippocampus
CP: cerebral peduncle
DARTEL: diffeomorphic anatomical registration through exponentiated lie algebra
DTI: diffusion tensor imaging
DWI: diffusion‐weighted images
EC: external capsule
EPI: echo planar imaging
FA: fractional anisotropy
FDR: false discovery rate
FWE: family‐wise error
GLM: general linear model
GM: gray matter
HCs: healthy control participants
HDL‐C: high‐density lipoprotein cholesterol
ICP: inferior cerebellar peduncle
ICV: intracranial volume
IFOF: inferior fronto‐occipital fasciculus
ILF: inferior longitudinal fasciculus
JHU: Johns Hopkins University
LDL‐C: low‐density lipoprotein cholesterol
MCP: middle cerebellar peduncle
ML: medial lemniscus
MMSE: Mini‐Mental State Examination
NDI: neurite density index
NODDI: neurite orientation dispersion and density imaging
ODI: orientation dispersion index
PCR: posterior corona radiata
PCT: pontine crossing tract
PLIC: posterior limb of internal capsule
PTR: posterior thalamic radiation
RD: radial diffusivity
ROI: regions of interest
SCP: superior cerebellar peduncle
SCR: superior corona radiata
SFOF: superior fronto‐occipital fasciculus
SLF: superior longitudinal fasciculus
TBSS: Tract‐based spatial statistics
TMT: Trail Making Test
TG: triglyceride
UF: uncinate fasciculus
VBM: voxel‐based morphometry
WM: white matter

## 1 Introduction

Dyslipidemia significantly contributes to atherosclerotic cardiovascular disease (Lukovits et al. 1999), characterized by reduced levels of serum high‐density lipoprotein cholesterol (HDL‐C) and increased levels of serum total cholesterol, low‐density lipoprotein cholesterol (LDL‐C), and triglycerides (TG). Studies have indicated that abnormal plasma lipid levels may influence cognitive function (Solomon et al. 2009; Segura et al. 2010). Interestingly, systemic cholesterol, particularly LDL‐C, can penetrate the brain (Dehouck et al. 1994; Dehouck et al. 1997; Karmi et al. 2010). This process is facilitated by receptors on the endothelial cells of the blood–brain barrier (BBB), serving as a channel for transporting LDL‐C from the circulation to the brain (Dehouck et al. 1994; Dehouck et al. 1997). The infiltration of LDL into brain tissue can lead to atherosclerosis or plaque formation within cerebral vessels (Boren et al. 2020). This process could lead to the degeneration of white matter (WM) due to a steady decrease in the circulation of oxygenated arterial blood (Williams et al. 2013). A previous study demonstrated a markedly increased likelihood of Alzheimer's disease or vascular dementia in individuals with atherosclerosis (Williams et al. 2013; Hofman et al. 1997). Dyslipidemia is a component of metabolic syndrome (MetS). Recent studies have demonstrated that MetS is associated with significant brain microstructural alterations, including reduced WM integrity in crucial brain regions (Shimoji et al. 2013; Andica et al. 2022), supporting the importance of evaluating the effect of serum lipid levels on WM microstructure. Furthermore, the mechanisms by which lipid dysregulation affects brain health are complex. A recent review emphasized the essential role of lipids, particularly cholesterol and phospholipids, in maintaining synaptic structure and function. For example, in Alzheimer's disease, disruptions in lipid metabolism can compromise synaptic integrity and induce microglial dysfunction (Paasila et al. 2021). However, dyslipidemia‐associated WM degeneration has not been fully investigated, and the underlying mechanisms of cognitive and motor dysfunction in individuals with dyslipidemia remain unclear.

The extensively employed method in studies to assess brain tissue microstructural changes is diffusion tensor imaging (DTI). Previous DTI studies have indicated WM microstructural alterations in individuals with abnormal cholesterol (Iriondo et al. 2021; Cohen et al. 2011). Notably, elevated levels of LDL‐C, total cholesterol, and TG were correlated with lower fractional anisotropy (FA) in WM areas related to executive functions and decision‐making. In addition, there was a positive association between LDL and axial diffusivity (AD) in posterior regions (Williams et al. 2013). Furthermore, elevated LDL‐C, HDL‐C, TG, and total cholesterol were significantly associated with higher AD in the corpus callosum (CC), corona radiata, superior longitudinal fasciculus (SLF), and internal capsule (Iriondo et al. 2021); however, there was no correlation with FA. This inconsistency may be due to DTI limitations. DTI indices, including FA, mean diffusivity (MD), AD, and radial diffusivity (RD), describe the magnitude of water molecule diffusion and the direction of diffusion (such as perpendicular and parallel to the axon) (Alexander et al. 2007). Although pathological conditions cause changes in various neuronal structures, such as the myelin sheath, axonal dispersion, density, diameter, and membrane permeability (Jones et al. 2013), the DTI model represents the diffusion behaviors related to these pathologies with a single tensor per voxel. Therefore, DTI has high sensitivity to evaluate changes in water molecule diffusion but lacks specificity for alterations in WM microstructure. Hence, it is challenging to deduce which neuronal structural changes are occurring by interpreting the DTI indices alone (Wheeler‐Kingshott and Cercignani 2009).

Neurite orientation dispersion and density imaging (NODDI) is a sophisticated neuroimaging modality, providing a more comprehensive assessment of WM microstructural properties compared with DTI. NODDI uses multi‐shell diffusion MRI data to differentiate between brain compartments (an intracellular compartment characterized by restricted anisotropic non‐Gaussian diffusion, reflected in the neurite density index [NDI]) and an extracellular compartment characterized by isotropic Gaussian diffusion represented by an isotropic volume fraction [ISO]) (Zhang et al. 2012). NODDI also calculates the orientation dispersion index (ODI), offering information on the directional distribution of neurites. In brief, NDI and ODI can be used to differentiate between the roles of axonal/dendritic density and fiber orientation, respectively (Spano et al. 2018). As far as we are aware, this is the first study to assess WM microstructure alterations in older individuals with dyslipidemia using NODDI.

The objective of this cross‐sectional research was to use NODDI to compare the WM microstructure between individuals with dyslipidemia and those without dyslipidemia and to investigate the link between these alterations and cognitive and motor function outcomes. We also compared the changes in DTI metrics and WM volumes between individuals with dyslipidemia and HCs. We hypothesized that using NODDI would provide a deeper insight into how dyslipidemia affects the microstructure of brain WM, in contrast to DTI.

## 2 Material and Methods

### 2.1 Study Participants

All study participants were enrolled in the Bunkyo Health Study (Someya et al. 2019), which comprises data from older adults in an urban community developed by the Sportology Center of Juntendo University. In November 2015, Juntendo University Hospital's ethics committee approved the study protocol (first approval no. 2015078 along with the most recent updated edition no. M15‐0057‐M08). All participants provided their written informed consent before their participation. This study adhered to the guidelines specified in the Declaration of Helsinki.

We included patients who had available and complete data for dyslipidemia‐related characteristics (LDL‐C, HDL‐C, and TG), neuropsychological measures, locomotor function tests, and brain 3‐T magnetic resonance imaging (MRI) data. We excluded participants with a history of substance or alcohol abuse, hypertension (systolic/diastolic blood pressures >140/90 mmHg or under treatment), diabetes mellitus (fasting plasma glucose level >126 mg/dL or under treatment), dementia (Mini‐Mental State Examination [MMSE] score of ≤23) (Ideno et al. 2012), depression (Japanese version of the 15‐item Geriatric Depression Scale [GDS‐15‐J] score of ≥10) (Sugishita et al. 2017), or any structural abnormalities on T1‐weighted brain images, such as brain tumors, arachnoid cysts, or vascular malformation, that could potentially affect registration steps in MRI analyses.

**FIGURE 1:**
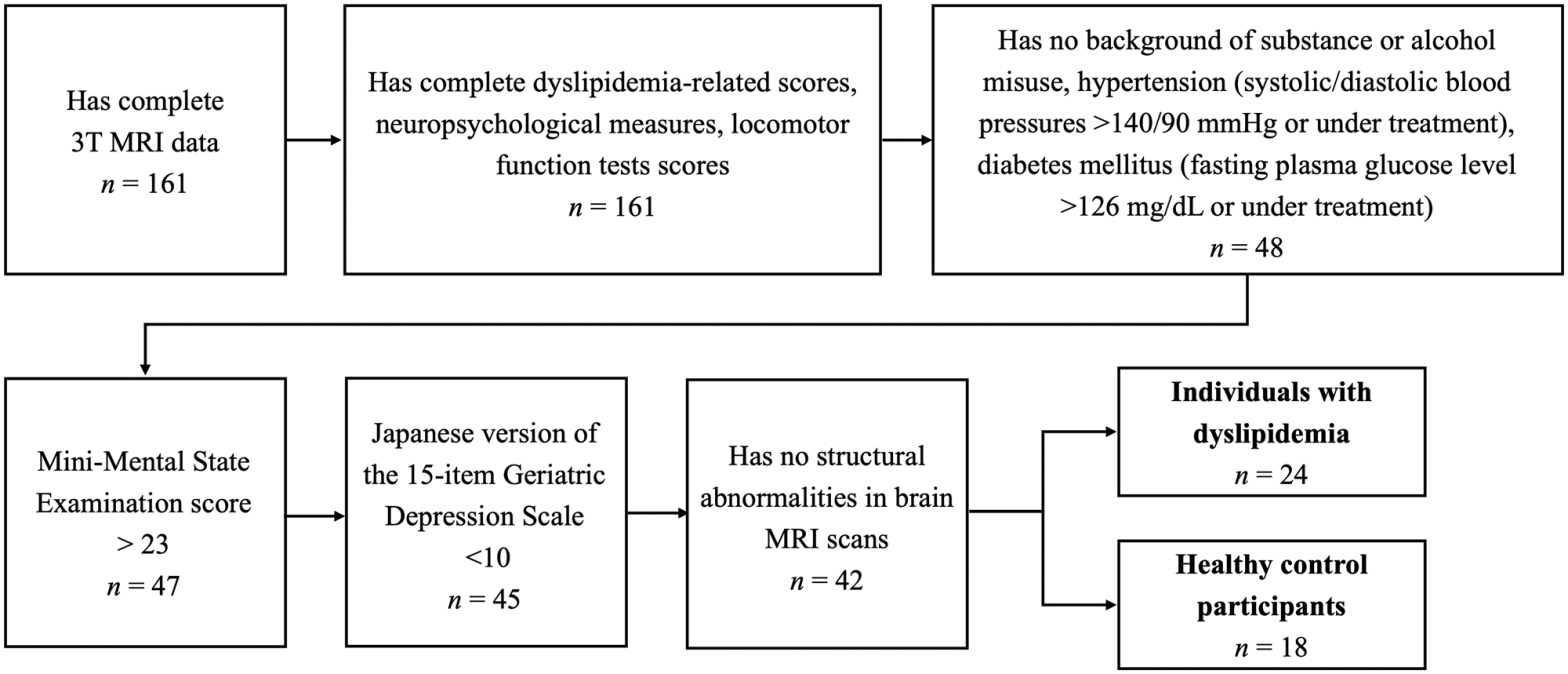
Participant eligibility flowchart. This chart includes the inclusion and exclusion criteria, along with the numbers of eligible and included participants.

Individuals diagnosed with dyslipidemia were categorized based on the Japan Atherosclerosis Society Guideline for the Diagnosis and Prevention of Atherosclerotic Cardiovascular Diseases 2017 (Kinoshita et al. 2018) for Japanese individuals, including the following three risk factors: (1) high levels of LDL‐C (≥140 mg/dL or undergoing dyslipidemia treatment), (2) low levels of HDL‐C (<40 mg/dL or undergoing treatment), and (3) elevated TG levels (≥150 mg/dL or ongoing lipid‐lowering medication usage). Dyslipidemia was diagnosed when any of these lipid abnormalities were present (Teramoto et al. 2007). The participants in the healthy control (HCs) group were age‐ and sex‐matched individuals with dyslipidemia who did not meet the aforementioned exclusion criteria and had no history of dyslipidemia (Figure 1). Table 1 summarizes the demographic and clinical profiles of individuals with dyslipidemia and HCs.

**TABLE 1:** Demographic characteristics of the study participants.

	HCs (*N* = 18)		Individuas with dyslipidemia (*N* = 24)		*P*‐value
	mean ± SD	Min–Max	mean ± SD	Min–Max	
Age (years)^a^	71.22 ± 4.68	66.00–82.00	71.08 ± 5.42	65.00–83.00	0.931
Sex (Male/Female)^c^	10/8		17/7		0.307
Years of education^a^	14.83 ± 2.00	12.00–18.00	14.13 ± 2.11	9.00–18.00	0.279
Handedness, *N* (left/right/mixed)^c^	(0/14/4)		(0/24/0)		**0.015**
BMI (kg/m^2^)^a^	21.49 ± 2.89	17.43–26.95	21.25 ± 2.48	16.09–25.48	0.775
*Dyslipidemia‐related characteristics*					
Total cholesterol (mg/dL)^a^	193.06 ± 25.92	148.00–238.00	237.67 ± 27.83	183.00–305.00	**<0.001**
HDL‐C (mg/dL)^a^	67.50 ± 10.28	50.00–91.00	63.67 ± 14.04	29.00–87.00	0.334
LDL‐C (mg/dL)^a^	109.56 ± 22.36	51.00–137.00	155.21 ± 25.57	99.00–223.00	**<0.001**
TG (mg/dL)^a^	70.50 ± 21.44	30.00–106.00	94.58 ± 34.99	48.00–180.00	**0.014**
Systolic blood pressure (mmHg)^a^	120.28 ± 10.76	104.00–136.00	122.42 ± 10.61	100.00–137.00	0.524
Diastolic blood pressure (mmHg)^a^	79.89 ± 6.19	71.00–89.00	77.91 ± 8.10	56.00–87.00	0.394
Fasted glucose (mg/dL)^a^	94.33 ± 10.04	74.00–117.00	94.54 ± 7.72	84.00–112.00	0.940
*Neuropsychological measures*					
MoCA‐J^a^	25.83 ± 2.64	21.00–30.00	26.00 ± 2.69	20.00–30.00	0.842
MMSE^a^	28.56 ± 1.34	26.00–30.00	28.17 ± 1.69	25.00–30.00	0.425
TMT‐A (sec)^a^	40.17 ± 9.04	25.00–63.00	42.71 ± 14.78	23.00–72.00	0.335
TMT‐B (sec)^a^	105.9 ± 40.52	59.00–225.00	94.96 ± 30.35	43.00–164.00	0.320
*Locomotor function tests*					
Stand‐up test^a^	4.44 ± 0.98	3.00–7.00	4.17 ± 0.96	2.00–6.00	0.365
Standardized two‐step test^a^	1.42 ± 0.16	1.03–1.59	1.40 ± 0.14	1.14–1.67	0.654
GLF‐S 25^a^	6.06 ± 7.23	0.00–31.00	3.29 ± 4. 62	0.00–19.00	0.139
*Fazekas scale*					
Total Fazekas scale^b^	2.33 ± 0.84	2–5	2.04 ± 0.69	0–3	0.542
Periventricular WM, *N* (0/1/2/3)^b^	(0/16/2/0)		(2/21/1/0)		0.161
Deep WM, *N* (0/1/2/3)^b^	(0/15/2/1)		(2/18/4/0)		0.546

Note: Data are presented as the mean ± standard deviation. Statistical analyses were performed using the unpaired Student's *t*‐test^a^, Mann‐Whitney *U* test^b^, or the χ^2^ test^c^. Statistically significant *P*‐values (<0.05) are written in bold.

Abbreviations: BMI, body mass index; GLF‐S 25, 25‐question geriatric locomotive function scale; HCs, healthy control participants; HDL‐C, high‐density lipoprotein cholesterol; LDL‐C, low‐density lipoprotein cholesterol; MMSE, Mini‐Mental State Examination; MoCA‐J, Japanese version of Montreal Cognitive Assessment; TG, triglyceride; TMT, Trail Making Test; WM, white matter.

### 2.2 Locomotor Function Tests

Locomotive function was assessed using the stand‐up test, the two‐step test, and the 25‐item geriatric locomotive function scale (GLF‐S‐25). The stand‐up test evaluates leg strength by having the subject stand up from seats of varying heights—40, 30, 20, and 10 cm—first with both legs, then with one leg. Subjects were considered to have passed if they could stand and maintain a given posture for three seconds. The inability to stand up on one leg from a 40 cm seat was considered a test failure. The two‐step test assesses walking ability by measuring the stride length over two steps, with the score calculated as the total stride length divided by the subject's height. The GLF‐S‐25 is a self‐administered questionnaire developed by Seichi et al. (Seichi et al. 2012) that evaluates pain, daily activities, social function, and mental health over the past month, with higher scores indicating worse locomotive function. A cutoff score of 16 suggests locomotive syndrome‐related disability. Detailed descriptions of these tests are available in previous studies (Someya et al. 2019; Yoshimura et al. 2015; Ogata et al. 2015).

### 2.3 MRI Acquisition

All MRI scans were acquired using a 3‐T MRI scanner (MAGNETOM Prisma; Siemens Healthineers, Erlangen, Germany) equipped with a 64‐channel parallel head coil. Three‐dimensional T1‐weighted images (3D‐T1WI) were obtained using magnetization‐prepared rapid gradient echo sequence with the following parameters: repetition time (TR) = 2,300 ms, echo time (TE) = 2.32 ms, inversion time = 900 ms, field of view (FOV) = 240 mm × 240 mm, matrix size = 256 × 256, slice thickness = 0.9 mm, voxel size = 0.9 mm × 0.9 mm × 0.9 mm, and acquisition time = 5 min 21 s. Diffusion‐weighted images (DWI) were obtained using spin‐echo planar imaging (EPI) in the anterior–posterior phase encoding direction at two *b*‐values of 1,000 and 2,000 s/mm^2^ (64 diffusion‐weighted directions, respectively), with one nondiffusion‐weighted (*b* = 0 s/mm^2^) volume using the following parameters: TR = 3,300 ms, TE = 70 ms, FOV = 229 mm × 229 mm, matrix size = 130 × 130, slice thickness = 1.8 mm, voxel size = 1.8 mm × 1.8 mm × 1.8 mm, and acquisition time = 7 min 29 s. Standard and reverse phase‐encoded blipped images (blip‐up and blip‐down) with no diffusion weighting were also acquired to correct for susceptibility‐induced distortions associated with EPI acquisition.

### 2.4 DWI Processing

All DWI data underwent processing via MRtrix3 (http://mrtrix.org) (Tournier et al. 2019) with the following steps: denoising (Veraart et al. 2016), Gibbs‐ringing artifact removal (Kellner et al. 2016), susceptibility‐induced distortion correction (Andersson et al. 2003), eddy current‐induced distortion, participant movements correction (Andersson and Sotiropoulos 2016), and B1 field inhomogeneity correction (Tustison et al. 2010).

We used the *DTIFIT* tool implemented in the FMRIB Software Library (FSL; Oxford Centre for Functional MRI of the Brain, Oxford, UK; http://www.fmrib.ox.ac.uk/fsl/) to obtain the DTI maps (FA, MD, AD, and RD) from single‐shell DWI data (*b* = 0, 1,000 s/mm^2^). These were based on a standard formula, according to Basser et al. (Basser et al. 1994). The DTI model assumes that water molecule diffusion in the tissue follows a Gaussian process (Basser et al. 1994; Basser and Jones 2002). However, at high *b*‐values (particularly values above 1,500 s/mm^2^), deviations from the Gaussian distribution due to tissue microstructure and compartmentalization can affect the diffusion tensor signal (Jensen et al. 2005). Therefore, in this study, we used only the *b* = 0 and 1,000 s/mm^2^ data to generate DTI maps.

The NODDI model was applied to the multi‐shell DWI data to obtain the NDI, ODI, and ISO maps (Zhang et al. 2012) using the accelerated microstructure imaging via convex optimization (AMICO) technique (Daducci et al. 2015).

### 2.5 Tract‐Based Spatial Statistics Analysis

Whole‐WM analyses were performed using tract‐based spatial statistics (TBSS) (Smith et al. 2006), which is a part of FSL (Jenkinson et al. 2012). First, FA images from all study participants were aligned with the mean FA standard template (FMRIB_FA) within a standard space of 1 × 1 × 1 mm, utilizing the FMRIB's Nonlinear Registration Tool (Jenkinson et al. 2012). Second, the mean FA image, derived from all aligned FA images, was skeletonized to form the mean FA skeleton that identified the most central and reliable WM tracts across all study participants. Subsequently, the primary WM tracts were included, and peripheral tracts, gray matter (GM), and CSF were excluded by setting the threshold of the mean FA skeleton to >0.2. Finally, the DTI (except for FA) and NODDI maps were aligned with the 1 × 1 × 1 mm standard space by applying the warpfield obtained from the initial registration steps and mapped onto the mean FA skeleton. Clusters exhibiting notable anatomical variances were pinpointed through the Johns Hopkins University (JHU) WM tractography atlas and the ICBM‐DTI‐81 WM labels atlas (Mori et al. 2009; Hua et al. 2008).

### 2.6 Region‐of‐Interest Analysis

Utilizing the JHU WM tractography atlas and the ICBM‐DTI‐81 WM labels atlas, the WM regions were segmented automatically and overlaid on the WM skeletons of the various participants. We then measured the mean values of DTI and NODDI indices in WM areas that differed significantly between individuals with dyslipidemia and HCs in the TBSS analysis.

### 2.7 WM Volumetry

WM volumetry was conducted using voxel‐based morphometry (VBM) through the Statistical Parametric Mapping (SPM) 12 software (https://www.fil.ion.ucl.ac.uk/spm/software/spm12/) on a MATLAB 2015b platform (The MathWorks, Natick, MA, https://www.mathworks.com/products/matlab.html) (Ashburner and Friston 2000). First, 3D‐T1WI underwent segmentation into GM, WM, and CSF based on standard tissue probability maps. Second, the Diffeomorphic Anatomical Registration Through Exponentiated Lie Algebra (DARTEL) algorithm was used to create a study‐specific template for spatial normalization of the segmented WM images of each study participant (Ashburner 2007). Finally, the segmented WM images were modulated using the Jacobean determinants of the deformation field obtained during spatial normalization with the DARTEL algorithm and were refined using a Gaussian kernel with an 8‐mm full width at half maximum. This step was adjusted for individual differences in brain size and preserved WM volumes with each voxel.

### 2.8 Statistical Analysis

All statistical analyses were performed utilizing IBM SPSS Statistics for Windows, version 22.0 (IBM Corporation, Armonk, NY, USA), with the exception of the general linear model (GLM) framework in the TBSS and VBM analyses. The mean and standard deviation were computed for each quantitative variable unless otherwise specified. The Shapiro–Wilk test was employed to evaluate the normality of the data. For all participants, demographic and clinical indices were analyzed using Student's *t*‐test or Mann‐Whitney *U* tests for normally or nonnormally distributed continuous variables and the χ^2^ test for categorical variables.

For TBSS analyses, a GLM framework, including age, sex, intracranial volume (ICV), and years of education as covariates, was employed to contrast the DTI and NODDI indices between individuals with dyslipidemia and HCs, utilizing the FSL randomize tool with 10,000 permutations (Jenkinson et al. 2012). The ICV was calculated by summing the volumes of GM, WM, and CSF obtained during the VBM analysis (Dale et al. 1999). All resulting statistical maps were modified to account for multiple comparisons by managing family‐wise error (FWE) and implementing threshold‐free cluster enhancement (Smith and Nichols 2009). An FWE‐corrected *P* value of <0.05 was considered statistically significant.

For VBM analyses, a GLM for analysis of covariance was employed to compare WM volumes between individuals with dyslipidemia and HCs. The covariates included age, sex, ICV, and years of education, with the FWE rate set at *P* = 0.05.

In the ROI analysis, a partial correlation test was performed separately in the dyslipidemia and HCs to assess the relationship between the NDI (which showed statistically significant group differences) and lipid profiles (total cholesterol, HDL‐C, LDL‐C, or TG) or locomotor function tests (stand‐up test, standardized two‐step test, and GLF‐S 25), adjusting for age, sex, and ICV. Additionally, the relationship between NDI and neuropsychological measures (MoCA‐J, MMSE, TMT‐A, or TMT‐B) were also performed while adjusting for age, sex, and ICV and the number of years of education. This analysis was performed on NDI values obtained from 30 WM regions, including the anterior thalamic radiation (ATR), anterior corona radiata (ACR), sagittal stratum (SS), anterior limb of internal capsule (ALIC), cerebral peduncle (CP), cingulum cingulate gyrus (CCG), middle cerebellar peduncle, cingulum hippocampus (CH), external capsule (EC), forceps major, forceps minor, inferior fronto‐occipital fasciculus (IFOF), genu, body, and splenium of CC, inferior longitudinal fasciculus (ILF), inferior cerebellar peduncle, tapetum, fornix, medial lemniscus (ML), posterior corona radiata (PCR), posterior thalamic radiation (PTR), pontine crossing tract, superior corona radiata (SCR), SLF, temporal part of SLF, superior fronto‐occipital fasciculus (SFOF), uncinate fasciculus (UF), superior cerebellar peduncle (SCP), posterior limb of internal capsule (PLIC), retrolenticular part of limb of internal capsule (RLIC), and fornix stria terminalis. The 30 selected ROIs have previously been shown to be implicated in studies evaluating the effects of abnormal serum lipid levels on regional WM microstructure (Williams et al. 2013; Andica et al. 2022; Ryu et al. 2017; Williams et al. 2019). The false discovery rate (FDR) method was employed to manage numerous comparisons across 30 ROIs for each correlation test between NDI and lipid profiles (i.e., total cholesterol, HDL‐C, LDL‐C, or TG), locomotor function tests (i.e., stand‐up test, standardized two‐step test, or GLF‐S 25), or neuropsychological measures (i.e., MoCA‐J, MMSE, TMT‐A, or TMT‐B). An FDR‐corrected *P* < 0.05 was considered statistically significant.

## 3 Results

### 3.1 Demographic and Clinical Assessments

Forty‐two older adults (18 HCs and 24 individuals with dyslipidemia) were enrolled in the current study. Age, sex, number of years of education, HDL‐C, systolic blood pressure, diastolic blood pressure, fasting plasma glucose, the Japanese version of the Montreal Cognitive Assessment (MoCA‐J) score, MMSE score, Trail Making Test parts A and B scores, stand‐up test score, the standardized two‐step test score, and the GLF‐S 25 score did not differ significantly between individuals with dyslipidemia and HCs. Individuals with dyslipidemia showed significantly elevated levels of total cholesterol (*P* < 0.001), LDL‐C (*P* < 0.001), and TG (*P* < 0.05) compared to HCs. Among participants with dyslipidemia, 62.50% (15/24) had a single abnormal blood lipid factor without medication, 16.67% (4/24) had one abnormal factor and were on medication, 8.33% (2/24) had two abnormal factors without medication, none had three, and 12.50% (3/24) had no abnormal lipid factors but were undergoing treatment for dyslipidemia.

### 3.2 TBSS Analysis

**FIGURE 2:**
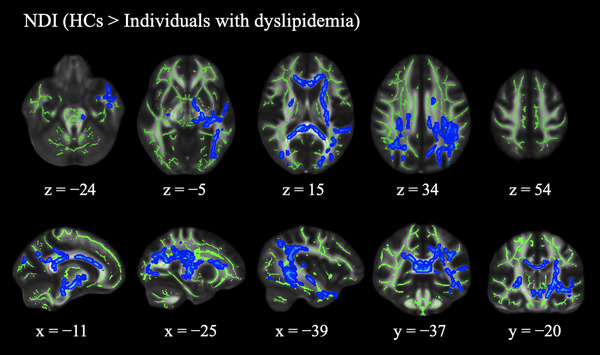
(A) NDI comparisons between healthy control participants and individuals with dyslipidemia. TBSS analyses reveal that individuals with dyslipidemia have significantly (*P* < 0.05, FWE‐corrected) reduced NDI (blue‐light blue voxels) compared to HCs. The mean FA skeleton is shown in green. *Abbreviations*: HCs, healthy control participants; TBSS, tract‐based spatial statistics; NDI, neurite density index.

According to our TBSS analysis, individuals with dyslipidemia exhibited significantly lower NDI than HCs (Figure 2). Reduced NDI was observed in multiple WM regions, including the bilateral CP, left SS, bilateral ALIC and PLIC, bilateral ACR, PCR and SCR, body, genu and splenium of CC, bilateral ATR and PTR, bilateral IFOF, fornix stria terminalis, bilateral corticospinal tract, bilateral RLIC, left ILF, left temporal part of SLF, bilateral SLF, tapetum, forceps major and minor, left SFOF, bilateral SCP, CCG, left UF, and left EC. No significant findings were observed in the ODI, ISO, and all DTI metrics. Supplemental Table 1 provides information on the anatomical regions, peak *t*‐value, and peak Montreal Neurological Institute coordinates of significant clusters.

### 3.3 Correlation Analysis

**FIGURE 3:**
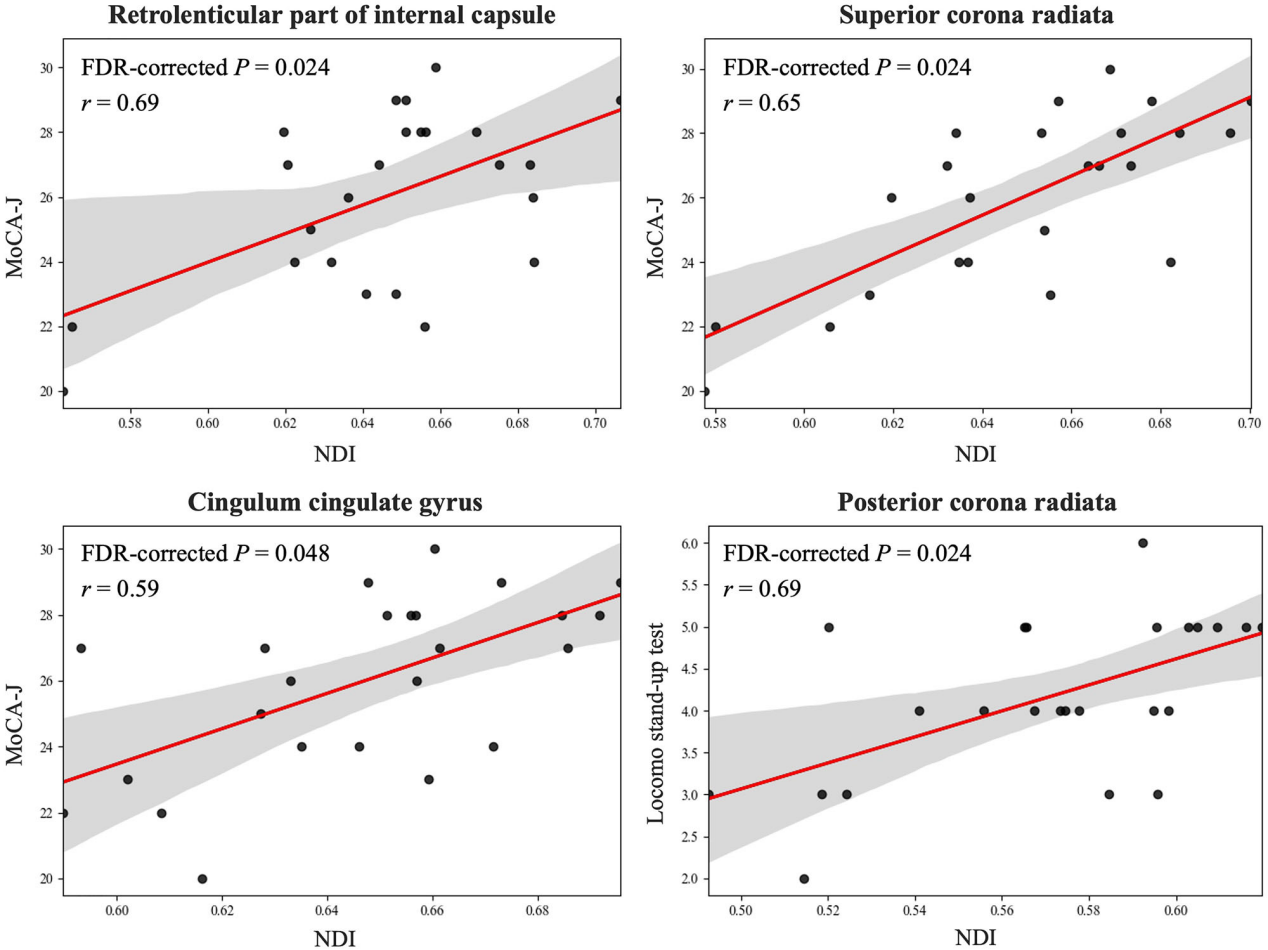
Partial correlation analyses, adjusting for age, sex, intracranial volume, and years of education (only for MoCA‐J), revealed significant positive correlations between NDI and the MoCA‐J or locomo stand‐up test. Red lines depict linear regressions with 95% confidence intervals (shadows). Abbreviations: NDI, neurite density index; MoCA‐J, Japanese version of Montreal Cognitive Assessment.

Figure 3 illustrates a notable positive link between NDI and MoCA‐J scores in the retrolenticular part of the internal capsule (*r* = 0.69, FDR‐corrected *P* = 0.024), SCR (*r* = 0.65, FDR‐corrected *P* = 0.024), and CCG (*r* = 0.59, FDR‐corrected *P* = 0.048) among individuals with dyslipidemia. Additionally, there was a significant positive correlation detected between NDI and the stand‐up test in PCR (*r* = 0.69, FDR‐corrected *P* = 0.024). However, no significant correlations were found between NDI and lipid profile component levels, such as LDL‐C, HDL‐C, TG, or total cholesterol. Furthermore, no significant findings were found within the HCs.

### 3.4 WM Volumetry

No notable disparities were observed in WM volumes between individuals with dyslipidemia and HCs.

## 4 Discussion

In this study, we applied NODDI to evaluate dyslipidemia‐related WM microstructural alterations and assess its association with cognitive and motor functions. Our major findings are as follows: First, significantly lower NDI was observed in individuals with dyslipidemia relative to HCs across WM regions. Conversely, the DTI metrics showed no notable disparities. In addition, significant positive associations were found between NDI and cognitive or motor function scores in specific WM areas.

### 4.1 Dyslipidemia‐Related Microstructural Changes in WM

Our study revealed that individuals with dyslipidemia manifested significantly reduced NDI levels compared with HCs, indicating lower intra‐axonal volume, which may suggest axonal loss (Zhang et al. 2012). A previous study indicated that dyslipidemia may result in increased lipid accumulation and lead to cell death, which in turn might cause neuronal degeneration in the hippocampus of apoE(−/−) dyslipidemia mouse models (Zhao et al. 2017). Similar to our findings, Ane Iriondo and colleagues’ research also revealed that plasma lipids are associated with axonal degeneration, as measured by neurofilament light chain and DTI (Iriondo et al. 2021). The potential mechanism for these findings may involve the oxidation of cholesterol in the brain, leading to the production of oxysterols such as 27‐hydroxycholesterol (Babiker et al. 2005; Harik‐Khan and Holmes 1990). Increased in vivo levels of 27‐hydroxycholesterol markedly diminish the levels of miR‐124a and miR‐9. MiR‐124 promotes neurite outgrowth and elongation (Gu et al. 2016). In addition, miR‐9 plays a crucial role in cortical circuit function, including neuronal axonal elongation and ramification. Furthermore, a decrease in miR‐9 levels is linked to hindered synaptic communication in the hippocampus (Dajas‐Bailador et al. 2012). Therefore, high concentrations of 27‐hydroxycholesterol disrupt neuronal branching and reduce synaptic density (Merino‐Serrais et al. 2019). In addition, recent studies have demonstrated that high MetS risk scores and obesity are associated with reduced myelin content, particularly in the prefrontal cortex, late‐myelinating regions, and areas associated with cognitive function (Burzynska et al. 2023; Bouhrara et al. 2021; Burzynska et al. 2024). 27‐Hydroxycholesterol, which crosses the blood–brain barrier, is toxic to immature oligodendrocytes (Alanko et al. 2023). Because oligodendrocyte function is essential for myelin maintenance and axonal support (Simons and Nave 2015), this may have significant implications for WM integrity. However, as diffusion MRI is not optimal for myelin content assessments, future studies should consider using myelin imaging techniques to evaluate dyslipidemia's impact on myelin. Combining NODDI with myelin imaging techniques could provide a more comprehensive understanding of dyslipidemia‐related pathologies.

Furthermore, dyslipidemia may be associated with disorders of the brain vascular system (Schilling et al. 2014). High levels of LDL‐C contribute to plaque accumulation in the cerebral arteries, which may induce a range of vascular issues (such as insufficient cerebral blood flow, blood–brain barrier damage, and thrombosis), potentially resulting in small capillary blockage. Such vascular disruptions can negatively affect neuronal health (Bang et al. 2005). This is supported by the findings from a chronic cerebral hypoperfusion mouse model, which showed neuronal cell loss in the cortex, segmental loss of fibers, and WM (Miki et al. 2009). Thus, the lower NDI in individuals with dyslipidemia that was captured in this study might reflect the pathological state of dyslipidemia.

Changes in NDI were predominantly noted in the left hemisphere. Although the reasons for this asymmetry remain unclear, several hypotheses have been considered, one of which is the direct anatomical linkage of the left carotid artery to the aortic arch, possibly resulting in high arterial pressure and, consequently, a higher susceptibility to atherosclerosis (Selwaness et al. 2014). A previous study using a chronic cerebral hypoperfusion model in mice revealed a greater decrease in cerebral blood flow on the left side compared to the right side within the context of a chronic cerebral hypoperfusion model by bilateral common carotid artery stenosis. Quantitative analyses of WM lesions also revealed that antineurofilament fiber densities in the left hemisphere were significantly reduced relative to those in the right hemisphere (Miki et al. 2009). Additionally, the predominance of right‐handed individuals warrants further investigation.

In contrast to the results of prior investigations indicating an inverse relationship between FA values and cholesterol levels in obese adults (Cohen et al. 2011), our findings showed no significant disparities in all DTI metrics between individuals with dyslipidemia and HCs. This could be attributed to the variances in the traits of the intended population. Our study revealed that the mean HDL‐C level in individuals with dyslipidemia (63.67 ± 14.04 mg/dL) was higher than that in the study performed by Cohen et al. (Cohen et al. 2011) in individuals with obesity (42.7 ± 1.6 mg/dL). Furthermore, this study revealed no significant disparity in HDL‐C levels between HCs and patients with dyslipidemia. It has been observed that HDL‐C has anti‐atherosclerotic properties (Perez‐Mendez et al. 2014). Furthermore, higher HDL‐C values might serve as a protective factor against WM hyperintensities (Wei et al. 2023). Moreover, every participant in our study was Japanese, in contrast to those in Cohen et al.’s study. The majority of the study participants were Caucasian and African American. Ethnic background is considered one of the risk factors for cardiovascular diseases (Grundy et al. 2018; Arnett et al. 2019). Nevertheless, taken together, our study might indicate that NODDI could facilitate the early detection of WM alterations, a finding that requires future validation via a longitudinal study. Furthermore, subsequent studies need to include people of different ethnicities to clarify the contribution of ethnicity to dyslipidemia‐related WM changes.

### 4.2 Associations Between Cognitive and Locomotor Dysfunctions

In individuals with dyslipidemia, a statistically significant correlation was found between lower NDI and lower MoCA‐J scores in the CCG, RLIC, and SCR. The cingulum is a crucial WM structure associated with emotion regulation, self‐awareness, and cognitive processes (Kobayashi 2011). Furthermore, the RLIC contains a group of WM fiber bundles implicated in motor control and coordination (Emos et al. 2023). SCR is crucial for transmitting sensory and motor nerve signals and integrating brain functions (Yin et al. 2018). Our findings indicate that damage to the RLIC and SCR could interrupt the flow of sensory and motor information, potentially leading to impaired cognitive processing.

Our findings also indicated a correlation between lower NDI and poorer performance on a locomotor stand‐up test in the PCR. The PCR provides the connection between the primary motor cortex and basal ganglia, which are crucial for motor control. Consequently, a reduction in axonal density within the PCR may disrupt the transmission of signals between these regions (Kwon et al. 2007), potentially leading to motor coordination problems. Overall, using NODDI, our findings showed that disorders in lipid metabolism may underlie cognitive and locomotor dysfunction in older adults, potentially due to axonal loss indicated by the lower intra‐axonal volume across several WM regions.

### 4.3 Study Limitations

A significant constraint of this research was its limited sample size and the restriction of the data to only cross‐sectional information. This is partly due to the high comorbidity of dyslipidemia with other vascular risk elements like hypertension and diabetes mellitus, which were part of our exclusion criteria. However, this limitation might also be considered one of this study's strengths, given that all our study participants were relatively unaffected by other conditions that tend to coexist with dyslipidemia and might also influence WM changes. Another limitation of this study was the unavailability of data on the duration of dyslipidemia among participants, which prevented us from exploring a potential association between disease duration and changes in WM. Future studies may benefit from the inclusion of this information or the assessment of longitudinal data to evaluate the potential impact of disease duration on WM alterations. Furthermore, some subjects in the dyslipidemia group were on medication. It is likely that the medication controlled dyslipidemia‐related profile values (HDL‐C, LDL‐C, and TG), as assessed by blood tests, which may explain why we did not find a statistically significant correlation between these values and NDI in the dyslipidemia group. However, the observed significant group differences in NDI might suggest that dyslipidemia treatment alone may not prevent neuronal changes in the brain associated with dyslipidemia. Future studies involving drug‐naïve individuals with dyslipidemia could be valuable for assessing the correlation between dyslipidemia‐related profiles and neurodegeneration.

## 5 Conclusions

According to our findings, we concluded that NODDI metrics are superior biomarkers of WM microstructural changes than DTI metrics in older individuals with dyslipidemia. Furthermore, our findings indicate that individuals with dyslipidemia experience axonal loss, indicated by lower intra‐axonal volume across a broad range of WM regions in contrast to HCs, and this loss is linked to cognitive and motor dysfunction. However, given this study's limitations, our findings should be interpreted cautiously when applied to clinical practice.

## Author Contributions

**Zaimire Mahemuti**: conceptualization, methodology, visualization, formal analysis, writing ‐ original draft. **Christina Andica**: conceptualization, methodology, formal analysis, writing ‐ review and editing. **Koji Kamagata**: conceptualization, formal analysis, methodology; writing ‐ review and editing. **Kaito Takabayashi**: conceptualization, formal analysis, methodology, writing ‐ review and editing. **Wataru Uchida**: visualization; writing ‐ review and editing. **Sen Guo**: visualization, writing ‐ review and editing. **Takashi Arai**: visualization; writing ‐ review and editing. **Hiroki Tabata**: conceptualization, methodology, supervision, writing ‐ review and editing. **Hitoshi Naito**: conceptualization, methodology, supervision, writing ‐ review and editing. **Yoshifumi Tamura**: conceptualization, methodology, supervision, writing ‐ review and editing. **Ryuzo Kawamori**: conceptualization, methodology, supervision, writing ‐ review and editing. **Hirotaka Watada**: conceptualization, methodology, supervision, writing ‐ review and editing. **Shigeki Aoki**: conceptualization, writing ‐ review and editing, supervision, funding acquisition.

## Ethics Statement

In November 2015, Juntendo University Hospital's ethics committee sanctioned the study protocol (first approval no. 2015078 along with the most recent updated edition no. M15‐0057‐M08). All participants in the study provided their written, informed agreement before their participation in the present research. This study adhered to the guidelines specified in the Declaration of Helsinki.

## Conflicts of Interest

The authors declare no conflicts of interest.

### Peer Review

The peer review history for this article is available at https://publons.com/publon/10.1002/brb3.70526

## Supporting information



Supporting Information

## Data Availability

The data supporting the findings of this study are available from the corresponding author upon reasonable request. The data are not publicly available due to privacy or ethical restrictions.
