# Supplementary material for: White Matter Microstructure Alterations in Older Adults With Dyslipidemia Associated With Cognitive and Locomotor Dysfunction Evaluated Using Neurite Orientation Dispersion and Density Imaging
Source: Brain Behav. 2025 May 28;15(6):e70526. doi: 10.1002/brb3.70526 (PMC12120193; doi:10.1002/brb3.70526)
Supplement: Supplementary file 1 — Supporting Information [file BRB3-15-e70526-s001.docx]

**Supplementary Table 1**. Tract-based spatial statistics analysis of NDI between individuals with dyslipidemia and healthy control participants.

| **Modality** | **Contrast** | **Cluster size** | **Anatomical region** | **Peak *t*-value** | **Peak MNI coordinates (*X*, *Y*, *Z*)** |
| --- | --- | --- | --- | --- | --- |
| NDI | HCs > Dyslipidemia | 12,387 | Bilateral CP, ALIC, PLIC, ACR, PCR, SCR, ATR, PTR, IFOF, corticospinal tract, RLIC, SLF, SCP; Lt-SS, ILF, temporal part of SLF, SFOF, UF, EC; tapetum, body, genu and splenium of CC; forceps major and minor, CCG and fornix stria terminalis | 5.19 | 69, 125, 84 |

*Abbreviations*: Lt, left; Rt, right; ACR, anterior corona radiata; ATR, anterior thalamic radiation; ALIC, anterior limb of internal capsule; CC, corpus callosum; CCG, cingulum cingulate gyrus; CP, cerebellar peduncle; EC, external capsule; HCs, healthy control participants; ILF, inferior longitudinal fasciculus; IFOF, inferior fronto-occipital fasciculus; LIC, limb of the internal capsule; MNI, Montreal Neurological Institute; NDI, neurite density index; PLIC, posterior limb of internal capsule; PCR, posterior corona radiata; PTR, posterior thalamic radiation; RLIC, retrolenticular part of internal capsule; SLF, superior longitudinal fasciculus; SCR, superior corona radiata; SCP, superior cerebellar peduncle; SFOF, superior fronto-occipital fasciculus; SS, sagittal stratum; UF, uncinate fasciculus.
